# An improved folate stable isotope dilution assay of unexploited food sources from Brazil

**DOI:** 10.3389/fnut.2023.1252497

**Published:** 2023-09-05

**Authors:** Lisa Obermaier, Barbara Paes Miglioli da Mata, Caio Humberto Perego, Kátia Sivieri, Mateus Kawata Salgaço, André Gonzaga dos Santos, Ruth Boehni, Viola Groehn, Jean-Pierre Knapp, Michael Rychlik

**Affiliations:** ^1^Chair of Analytical Food Chemistry, Technical University of Munich, Munich, Germany; ^2^Laboratory of Pharmacognosy, School of Pharmaceutical Sciences, São Paulo State University, São Paulo, Brazil; ^3^Merck & Cie KmG, Schaffhausen, Switzerland; ^4^Centre for Nutrition and Food Sciences, The Queensland Alliance for Agriculture and Food Innovation, The University of Queensland, Brisbane, QLD, Australia

**Keywords:** stable isotope dilution assay, LC–MS/MS, folate, unexploited Brazilian food crops, vitamer, validation

## Abstract

Brazil has a diverse plant community, including underutilized non-conventional food crops (PANCs), which have the potential to be a rich source of food and contribute to food security. For assessing the folate content in a range of Brazilian PANCs, we extended the validation of an existing stable isotope dilution assay (SIDA) for the stably ^13^C-labelled 10-formyl-Pte[^13^C_5_]Glu (10-CHO-Pte[^13^C_5_]Glu). The SIDA method with an enzymatic treatment, purification step, and an LC–MS/MS measurement was validated regarding linearity, precision, LoD/LoQ, and recovery for 10-CHO-PteGlu. After successful validation, the study of some underutilized Brazilian non-conventional fruits and leaves from the São Paulo State University campus revealed them as an important source of folates. It provided the first insights into the folate content of unexploited food sources from Brazil. Pequi had the highest folate content among the fruits studied, with mean values of 333 μg/100 g based on fresh weight (FW). The analysis also shows that different cultivars of fruit or fruits from different growing locations have a high variability in folate content or other nutritional factors. In most fruits, the main vitamer was 5-CH_3_-H_4_folate, but jenipapo and taioba showed the highest content of 10-CHO-PteGlu with 28.22 μg/100 g (FW) in jenipapo peel and 75.64 μg/100 g (FW) in the taioba leaves. Thus, this study also provides results on the importance of the folate vitamer 10-CHO-PteGlu contributing to the total folate content.

## 1. Introduction

The world’s population is projected to reach nearly 10 billion by 2050, and with this growth comes the need for increased food production and sources. However, as we strive to meet this demand, we must also consider the nutritional value of the food we produce and consume (1). Micronutrients, such as vitamins and minerals, are essential for human health and development, yet deficiencies in these nutrients are a widespread problem, particularly in low-and middle-income countries (2).

In Brazil, a country with diverse plant life, many plants still need to be exploited for food sources - around 90% remain underused (3). In this regard, the term PANCs stands for “Plantas Alimentícias Não Convencionais” in Portuguese, which means “Non-Conventional Food Plants.” It refers to plants that are not commonly produced, purchased, or even consumed as food but have the potential to be used as a rich source of nutrition. These plants can include wild or semi-domesticated species, traditional crops, and invasive species. PANCs can play a vital role in food security in rural and remote areas, where access to a diverse range of food sources is limited, even in large urban centers, where food prices can inflate a lot. One solution to this problem is to focus on increasing the availability of micronutrients through natural sources. This can be achieved by promoting locally sourced, nutrient-rich foods, such as leafy greens and fruits (4, 5). Some PANCs can be harvested free of charge in the streets, ornamental plant beds, rural and forest areas, and other places not belonging to the conventional agricultural matrix, facilitating their access and consumption. Many PANCs have mineral and vitamin levels higher than those found in traditional food vegetables (6, 7), which is a valuable contribution to diets and reinforces the beneficial potential of non-conventional plants. The dissemination and popularization of the nutritional value of native PANCs serve not only as nutritional and gastronomic benefits but also as a strategy for safeguarding biodiversity and traditional Brazilian culture.

One of the crucial micronutrients worldwide is folate. This water-soluble B_9_ vitamin plays an essential role in cell renewal and cell growth. It functions as a coenzyme in the metabolism of one-carbon groups and DNA synthesis (8). Folates cannot be synthesized *de novo* by the human organism and must therefore be ingested from external sources (9). Covering the recommended daily amount of folates is particularly important for pregnant women and women of childbearing age since folate deficiency increases the risk of developing neural tube defects and other malformations in the fetuses. Besides, folate deficiencies are associated with an increased risk for cardiovascular diseases, colorectal cancer, and Alzheimer’s disease (10).

The folate deficiency situation in Brazil has improved since flour fortification with folic acid became mandatory in 2004. To date, wheat and corn flour are fortified with 150 μg PteGlu/100 g flour, resulting in an average daily folate intake of 376 μg in the studied population in 2014 (11). Yet fortification is done with folic acid, which is suspected of having a deleterious effect on vascular walls and increasing the risk of colon and prostate cancer and cognitive impairment with excessive intake (12). And processed foods made from enriched wheat flour contribute significantly to the folate intake (11). Given these profound consequences of a deficiency on one side and an excess of folic acid (via supplements) on the other, it becomes important to discover natural folate sources.

Therefore, a reliable and sensitive analytical method for the exact quantification of the folate content in food is to provide reasonable consumption recommendations for adequate folate supply. A stable isotope dilution analysis (SIDA) coupled with an LC–MS/MS measurement has proven to be the method of choice for folate quantification over the past few years (13–16). SIDA uses stably isotope-labeled analogues that have almost identical chemical and physical properties as the analyte to be quantified. Due to the compensation for losses and interferences caused by matrix components in sample clean-up and mass spectrometric analysis, precise quantification is possible (17). Striegel et al. (18) improved and validated this method in respect of four folate vitamers, namely pteroylmonoglutamic acid (PteGlu), tetrahydrofolate (H_4_folate), 5-methyltetrahydrofolate (5-CH_3_-H_4_folate), and 5-formyl-tetrahydrofolate (5-CHO-H_4_folate) with strawberries as a test matrix. This study deals with the validation of one “missing” folate vitamer, namely 10-formyl-folic acid (10-CHO-PteGlu), regarding linearity, precision, recovery, the limit of detection (LoD), and limit of quantification (LoQ) according to (19). 10-CHO-PteGlu occurs as the main vitamer in, e.g., cereal grains and some vegetables like Choy Sum (20, 21), can contribute to the total folate content of some food sources and therefore, is essential to quantify accurately. The test matrix used in this validation are unexploited Brazilian PANCs, including fruit peel, pulp, and leaves. We intend to demonstrate that high levels of 10-CHO-PteGlu are present in some food sources, contributing to the overall folate content. This estimation is based on our prior research findings. Previously, the content of this vitamin could only be determined using the isotopically labeled standard of 5-CHO-H_4_folate vitamer as an internal standard. The corresponding isotopically labeled standard 10-CHO-Pte[^13^C_5_]Glu is much more similar to the corresponding analyte. Therefore, losses during workup and measurement could be more accurately accounted for. Apart from that, the data of this study are intended to provide first insights into the folate content of unexploited food sources from Brazil.

## 2. Materials and methods

### 2.1. Sample

The fruits or leaves of all plant species were collected from specimens located at the campus of the São Paulo State University (Araraquara, São Paulo, Brazil, −21.81448558950186 S, − 48.19835063373754 W, April 2019). For further analysis of the pequis additional fruits were collected in a rural area of the same city as the first two pequis (1 and 2) (−21.66908776818103 S, −48.245748264360614 W, February 2019, pequi 4) and purchased (commercial pequi fruit, peeled and frozen) from Goiânia (Goiás, Brazil, −16.68581680501811 S, − 49.265932078447186 W, pequi 3). Two other ones from the same specimen as pequi sample 4, just on a new crop, were also analyzed (pequi 5 and 6).

The pulp and peel of the fruits and the leaves from the same cultivar were combined, stored at – 80°C, freeze-dried, and milled in an analytical mill (Ika®, model A11). The samples aliquots were again stored at - 80°C and randomly selected for further analysis. The residual moisture was determined with one aliquot by loss on desiccation with infrared radiation (Gehaka®, model IV 2000). This study was registered under the codes R785DF1 and RD56E84 of the Brazilian National System for the Management of Genetic Heritage and Associated Traditional Knowledge (SisGen). As the samples were freeze-dried prior to the analysis, the water content from the literature was considered, and the folate concentrations were calculated on a fresh weight basis.

### 2.2. Sample set

Peel and pulp of *Garcinia brasiliensis* Mart. (Bacupari), peel and pulp of *Pouteria campechiana* (Kunth) Baeni (Canistel), peel and pulp of *Hymenaea courbaril* L. (Jatobá), peel and pulp of *Genipa americana* L. (Jenipapo), peel and pulp of *Licania tomentosa* (Benth) Fritsch (Oiti), external and internal pulp of *Caryocar brasiliense* Cambess. (Pequi), leaves of *Pereskia aculeata* Mill. (Ora-pro-nóbis), leaves of *Xanthosoma sagittifolium* (L.) Schott (Taioba), and leaves of *Varronia curassavica* Jacq. (Erva-baleeira). Further information on the samples and quantities of fruit cultivars we used can be found in Supplementary Table 1.

#### 2.2.1. Reagents, standards, and enzymes

LC–MS grade acetonitrile (ACN), methanol (MeOH), and water were purchased from VWR (Ismaning, Germany); ascorbic acid, formic acid (> 95%), and 2-(N-morpholino)-ethane sulfonic acid (MES) were purchased from Sigma-Aldrich (Steinheim, Germany); potassium dihydrogen phosphate, sodium acetate trihydrate, and sodium hydroxide were purchased from Merck (Darmstadt, Germany); disodium hydrogen phosphate (anhydrous), and sodium chloride was obtained from Alfa Aesar and Baker J.T. (Thermo Fisher, Karlsruhe, Germany); rat serum and chicken pancreas containing γ-glutamyl hydrolase (EC 3.4.19.9) were purchased from Biozol (Eching, Germany) and Difco (Sparks, MD, USA), respectively; and Dithiothreitol (DTT) from AppliChem (Darmstadt, Germany). The unlabelled reference compounds ((6S)-H_4_folate, (6R,S)-5-CH_3_-H_4_folate, (6R,S)-5-CHO-H_4_folate, PteGlu and, 10-CHO-folate) were purchased from Schircks Laboratories (Jona, Switzerland), the isotopological internal standards (Pte[^13^C_5_]Glu, (6S)-H_4_Pte[^13^C_5_]Glu, (6S)-5-CH_3_-H_4_Pte[^13^C_5_]Glu-Ca and (6S)-5-CHO-H_4_Pte[^13^C_5_]Glu-Ca) were obtained from Merck & Cie KmG (Schaffhausen, Switzerland) The stably labeled folate vitamer 10-CHO-Pte[^13^C_5_]Glu to investigate the folate vitamer 10-CHO-PteGlu in PANCs was provided by Merck & Cie KmG (Schaffhausen, Switzerland), as well. The Strata SAX cartridges (quaternary amine, 500 mg, 3 mL) for the solid phase extraction were acquired from Phenomenex (Aschaffenburg, Germany).

For the extraction, a buffer containing 2 g/L ascorbic acid and MES (200 mmol/L) solution with 0.1 g DTT, adjusted to pH 5 with 7.5 M NaOH, was used. To dissolve the folate standards and as an equilibration buffer for the SAX cartridges, a phosphate buffer (100 mmol/L) was prepared. Therefore, 100 mmol/L disodium hydrogen phosphate aqueous solution was adjusted to pH 7.0 with a potassium dihydrogen phosphate (100 mmol/L) aqueous solution. The equilibration buffer was prepared with 0.2 g/L DTT and 10 mmol/L phosphate buffer and adjusted to pH 5 with NaOH. The elution buffer consisted of 5% aqueous sodium chloride, 100 mmol/L aqueous sodium acetate, 0.1 g DTT, and 1% ascorbic acid.

For the enzymatic treatment, the chicken pancreas conjugase solution was prepared by adding 30 mg lyophilized chicken pancreas to 30 mL aqueous phosphate buffer solution (100 mmol/L) and 1% ascorbic acid adjusted to pH 7. Additionally, the purchased rat serum was thawed once, and both enzyme solutions were treated with activated carbon for 30 min and filtered with 0.45 μm molecular filters.

For the stock solutions of the reference compounds, 10 mg of PteGlu was dissolved in 10 mL phosphate buffer and filled up to 100 mL with extraction buffer. For the other reference standards, 2 mg of H_4_folate, 5-CH_3_-H_4_folate, 5-CHO-H_4_folate, and 10-CHO-PteGlu were dissolved in 3 mL phosphate buffer and filled up to 10 mL with extraction buffer, respectively. The exact concentration of the freshly prepared, unlabelled analytes was determined within every extraction day with HPLC-DAD. Therefore, PteGlu is used as the internal standard (IS) for the quantification of 5-CH_3_-H_4_folate, 5-CHO-H_4_folate, and 10-CHO-PteGlu, and 5-CH_3_-H_4_folate is used as the internal standard for the determination of H_4_folate. For the LC–MS/MS response, the stock solutions were further diluted to 1:20 and 1:10 for PteGlu. The labeled standards [^13^C_5_]-PteGlu, [^13^C_5_]-H_4_folate, [^13^C_5_]-5-CH_3_-H_4_folate, [^13^C_5_]-5-CHO-H_4_folate and [^13^C_5_]-10-CHO-PteGlu were once dissolved in concentrations of 60–70 μg/mL in extraction buffer and further diluted to a final concentration of 8–11 μg/mL. The labeled reference solutions were stored at -20°C in the dark.

#### 2.2.2. Sample extraction and purification

The preparation of the buffers, the stock solutions, enzymes, and the extraction was done as described by Striegel et al. (18) with slight modifications.

All steps of sample extraction and purification were conducted under subdued light. The homogeneous sample (appr. 20 mg) was equilibrated in 10 mL of extraction buffer with a magnetic stirrer for 15 min. To perform the SIDA, the expected content of the respective analytes was evaluated, and an approximately similar amount of labeled standard was added to the sample. After another 15 min of equilibration, the samples were boiled in a water bath for 10 min and cooled on ice. Each sample was treated with 900 μL chicken pancreas and 200 μL rat serum and afterwards incubated overnight (min. 6 h) at 37°C in a shaking bath. After incubation, the samples were again boiled for 10 min. The cooled extracts were transferred in 50 mL-centrifuge tubes with 10 mL ACN and centrifuged for 20 min at 4°C (4,000 rpm). The supernatant containing the organic and aqueous phases was purified using solid phase extraction with a strong anion exchange cartridge. Before purification, the cartridges were conditioned with two volumes of MeOH and equilibrated with two volumes of equilibration buffer. After loading the cartridges with the sample, they were washed with two volumes of equilibration buffer. The elution of the folates was conducted with 4 mL elution buffer. The purified samples were membrane filtered (PVDF, 0.22 μm) and measured on the LC–MS/MS system. Besides the samples, a response mixture for all analytes and internal standards and the blank was measured. For the blank, 10 mL extraction buffer was spiked with 5 μL of each labeled standard and prepared like the samples. The endogenous number of folates in each sample was evaluated by subtracting the folate content of the chicken pancreas and the rat serum determined in the blank measurement.

#### 2.2.3. HPLC– and LC–MS/MS methods

The analysis was performed using (18) previously published instrumental conditions with slight modifications.

The purity check of the unlabelled reference solutions was conducted on a Shimadzu HPLC/DAD system (Shimadzu, Kyoto, Japan) using a reversed-phase column (C18 EC, 250 × 3 mm, 5 μm, 100 Å, precolumn: C18, 8 × 3 mm, Machery-Nagel, Düren, Germany). 10 μL of the sample were injected, and the column oven temperature was set to 25°C. The mobile phases were (A) 0.1% acetic acid and (B) methanol. The analysis was performed at a flow rate of 0.4 mL/min. The gradient started at 10% B. After 7 min equilibration time, the concentration of B was linearly raised to 50% within the next 14 min. Then, the gradient linearly went up to 100% B within 2 min and held at 100% B for 1 min. Finally, the mobile phase returned to the starting condition (10% B) within 2 min and was equilibrated for 9 min before the next run.

For the LC–MS/MS measurement, a Shimadzu Nexera X2 UHPLC system (Shimadzu, Kyoto, Japan) with a Raptor ARC-18 column (2.7 μm, 100 mm × 2.1 mm, Restek, Bad Homburg, Germany) and a Raptor ARC-18 precolumn (2.7 μm, 5 mm × 2.1 mm, Restek, Bad Homburg, Germany) as a stationary phase was used. The injection volume was 10 μL, and the separation was conducted at 30°C.

The mobile phases were (A) 0.1% formic acid and (B) acetonitrile with 0.1% formic acid. The analysis was performed at a flow rate of 0.4 mL/min. The gradient elution started at 3% B, was linearly raised to 10% B within the next 2.5 min, and was held for 2.5 min. Then, the gradient linearly went up to 15% B within 5 min and to 50% during a further 1 min, where it maintained for 1 min. Subsequently, the gradient returned to the starting condition (3% B) for 4 min.

The triple quadrupole mass spectrometer (LCMS-8050, Shimadzu, Kyoto, Japan) was operated in the positive ESI mode for all analytes. The ion source parameters were optimized prior to this study by injecting each labeled and unlabelled standard solution (1 mg/L). The heat block, dilution line, and interface temperature were maintained at 400°C, 250°C and 300°C, respectively; drying gas, heating gas, and nebulizing gas flow were set to 10 L/min, 10 L/min, and 3 L/min, respectively, collision-induced dissociation gas was applied to 270 kPa, and interface voltage was applied to 4 kV. The column effluent was only diverted to the mass spectrometer from 2.1 to 7.0 min. The data was acquired using multiple reaction monitoring (MRM). The detailed conditions are described in Supplementary Table 2.

The measurements were analyzed and evaluated with the LabSolutions software 5.91 (Shimadzu, Kyoto, Japan).

### 2.3. Statistical analysis

The analysis was performed in triplicates (with triplicate injection) and calculated, including ± standard deviation (RSD, %), shown as error bars in the following figures. As the data of the samples are only indicative and not representative of the sample number and sample size, we did not perform statistical tests for significant differences.

### 2.4. Validation

For the validation of the LC–MS/MS method for the 10-CHO-PteGlu vitamer, twelve calibration points with constant amounts of internal standard (IS) and varying amounts of analyte (A) from 0.87 μg/L to 261.6 μg/L were measured as a response curve. The molar ratios [n(A)/n(IS)] were between 0.01 and 15.20. Linear regression analysis was performed by combining the molar ratios [n(A)/n(IS)] with the peak area ratios [A(A)/A(IS)] from the LC–MS/MS measurements. To confirm the linearity, Mandel’s test was conducted (Mandel 1964).

The Limits of Detection and Quantification (LoD, LoQ) were determined according to (19), where the LoD is three times the signal-to-noise ratio. Respectively the LoQ is ten times higher than the value obtained from the blank analysis. In this study, the blank matrix was a recombinant consisting of starch (for analysis) and sugar in a 3:1 ratio, as it should represent a magnitude of fruits and vegetables. To reduce the folates present in the starch, the matrix was treated under UV light and analyzed to evaluate the left folates. To determine the LoD and LoQ, the matrix was spiked with the unlabelled analyte (10-CHO-PteGlu) at five different amounts. The extraction work-up was done in triplicate for each level. The lowest concentration was slightly above the estimated LoD, and the highest was tenfold higher. At each level, the labeled standards were equal to 0.01 nmol. The correlation of the labeled standard and the analyte and a regression calculation provided a new, expanded calibration line. Besides, it showed the prediction intervals used to determine the LoD and LoQ (19).

To evaluate the precision of the measurement regarding the labeled analyte, intra-day, inter-day, and inter-injection precision measurements were performed. Therefore, one of the analyzed samples, Jenipapo pulp, was used. The inter-injection precision was determined with one sample (*n* = 1) injected 12 times in a row (*i* = 12). One sample was analyzed in triplicate within three independent extractions (*n* = 9) and triplicate injections (*i* = 27) over 3 weeks to determine the inter-day precisions. The intra-day precision was calculated with one sample in a triplicate extraction (*n* = 3) and triplicate measurement (*i* = 9).

To determine the recovery, a blank matrix was spiked in triplicate with 10-CHO-PteGlu in three different concentrations, and the standard work-up was conducted. The three spiking levels for the recoveries were 0.92 μg/100 g (low), 21.27 μg/100 g (medium), and 170.55 μg/100 g (high). Due to the ubiquitous presence of folates in food, we used a self-composed mixture as the surrogate for a blank matrix. Therefore, we used a 3:1 mixture of saccharose and starch depleted from folates by treatment with UV light. But as all analytes could still be detected in small amounts, they had to be subtracted from the spiked matrices. The recoveries were calculated as the ratio of the detected and spiked contents, whereas the values should be between 70 and 120%, according to (19).

## 3. Results and discussion

### 3.1. Validation

Linear regression resulted in a calibration curve for 10-CHO-PteGlu with *y* = 1.2446x – 0.0054. The curve revealed proven linearity with Mandel’s test. The linear range of area ratios ranged between 0.06 and 11.56, with a coefficient of determination of 0.999.

**Figure 1:**
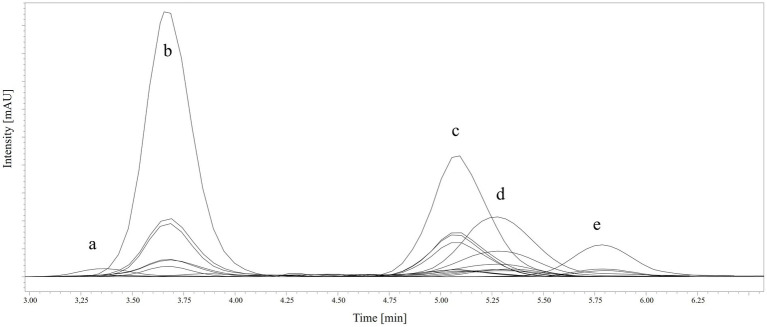
LC–MS/MS-chromatogram of a pequi sample with the five vitamers and the respective internal standards [(a) H_4_folate, [^13^C_5_]-H_4_folate; (b) 5-CH_3_-H_4_folate, [^13^C_5_]-5-CH_3_-H_4_folate; (c) 10-CHO-PteGlu, [^13^C_5_]-10-CHO-PteGlu; (d) 5-CHO-H_4_folate, [^13^C_5_]-5-CHO-H_4_folate; (e) PteGlu, [^13^C_5_]-PteGlu].

In comparison, the validation of the four other vitamers by Striegel et al. (18) showed a polynomial regression line for 5-CH_3_-H_4_folate and 5-CHO-H_4_folate between 0.03 and 9.11, respectively 10.10 for 5-CH_3_-H_4_folate and linear regressions between 0.10 and 14.90 for PteGlu and H_4_folate (see Table 1). Figure 1 shows a complete chromatogram of a sample (pequi) with the five different vitamers and their respective internal standards.

**Table 1:** LoDs and LoQs for validation by Striegel et al. (18) and the new validation of 10-CHO-PteGlu (marked with *).

Analyte	LOD [μg/100 g]	LOQ [μg/100 g]	Precision [RSD, %]^1^			Recovery [%]		
			Inter-injection	Intra-day	Inter-day	Spiking level 1 (0.92 μg/100 g)	Spiking level 2 (21.27 μg/100 g)	Spiking level 3 (170.55 μg/100 g)
PteGlu	0.33	0.96	3.82^2^	2.70	4.83^2^	109	114	105
H_4_Folate	0.25	0.76	4.46	2.44	5.06	99.1	99.4	99.3
5-CH_3_-H_4_Folate	0.17	0.51	1.92	2.74	3.04	96.7	99.5	101
5-CHO-H4Folate	0.32	0.93	2.49	4.60	4.83	81.9	98.2	96.7
10-CHO-PteGlu^*^	0.28^*^	0.85^*^	3.47^*^	1.90^*^	7.95^*^	112.5 ± 11.2^*^	90.2 ± 9.1^*^	93.1 ± 9.9^*^

1 For determination of the precisions, an actual sample was used.

2 For determination of the inter-day and intra-day precisions, a matrix of sugar and pectin was used.

The validation provided an LoD of 0.28 μg/100 g and LoQ of 0.85 μg/100 g for 10-CHO-PteGlu. The validation results of the four other vitamers showed slightly higher LoDs for PteGlu and 5-CHO-H_4_folate and lower levels for H_4_folate and 5-CH_3_-H_4_folate compared to our previous validation (18) (see Table 1). The detection limit of 10-CHO-PteGlu lies between those groups.

Table 1 shows the validation results regarding precision and recovery. One of the PANC samples was used to determine the precisions. The relative standard deviations from 1.9 to 7.95% presented here were similar or even better than those previously reported for SIDAs (14) and in the same range as the precisions occurred in the validation of the other four vitamers (18).

The recovery in this study for the 10-CHO-PteGlu ranged between 90.2 and 112.5%, which lies in the same range as previously reported for strawberries. These results proved this method’s reliability and sensitivity, including the vitamer 10-CHO-PteGlu.

### 3.2. Folate content and vitamer distribution

**Figure 2:**
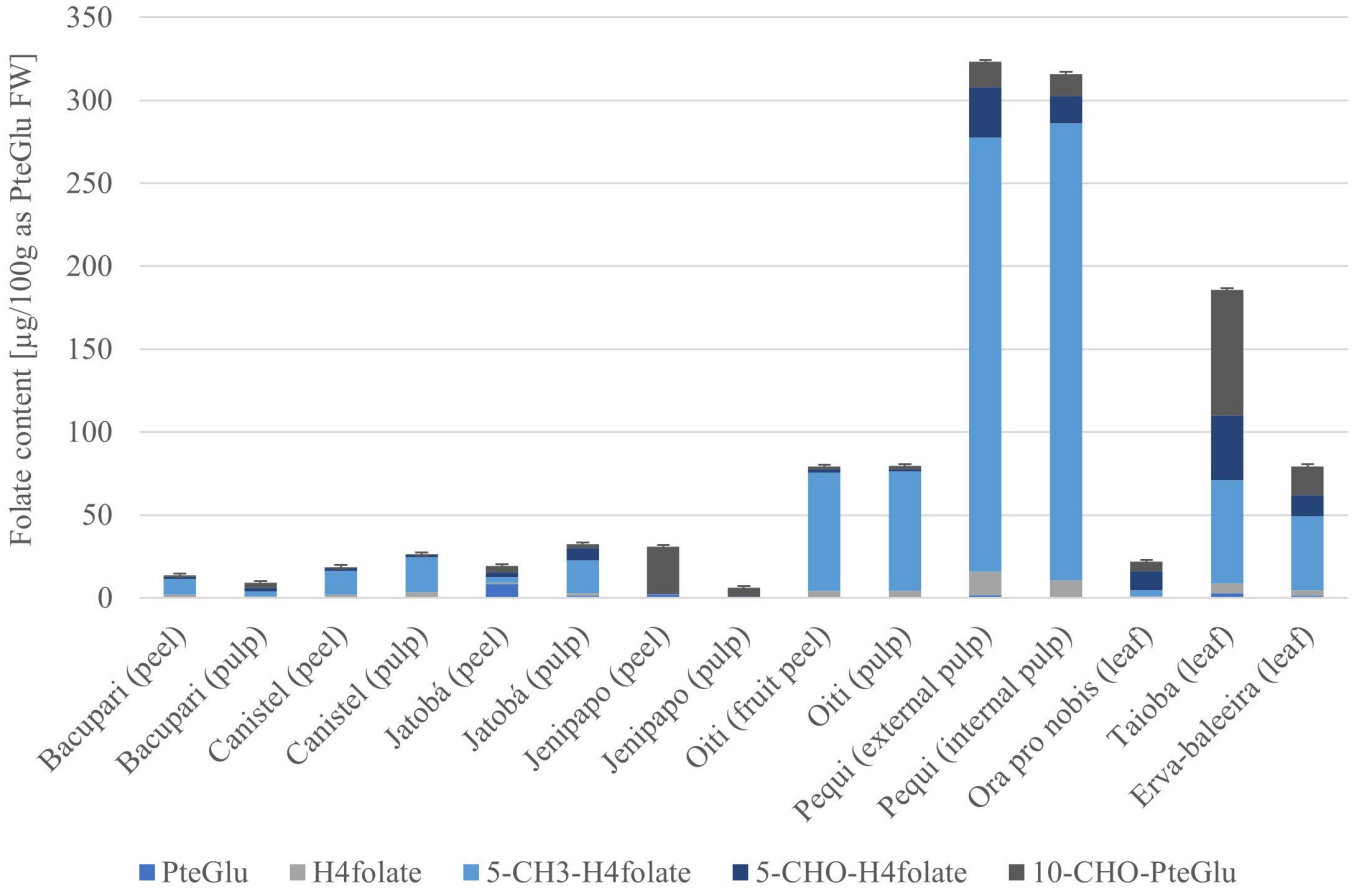
Vitamers distribution of the PANCs, calculated as PteGlu in [μg/100 g], based on fresh weight (FW), error bars show relative standard deviation.

Figure 2 show that half of the analyzed PANCs have a total folate content of less than 33 μg/100 g based on fresh weight (FW). The outstanding fruit was pequi, both internal and external pulp, with more than 300 μg/100 g FW content, and the Taioba leaves with 185 μg/100 g FW (values in Supplementary Table 3).

**Figure 3:**
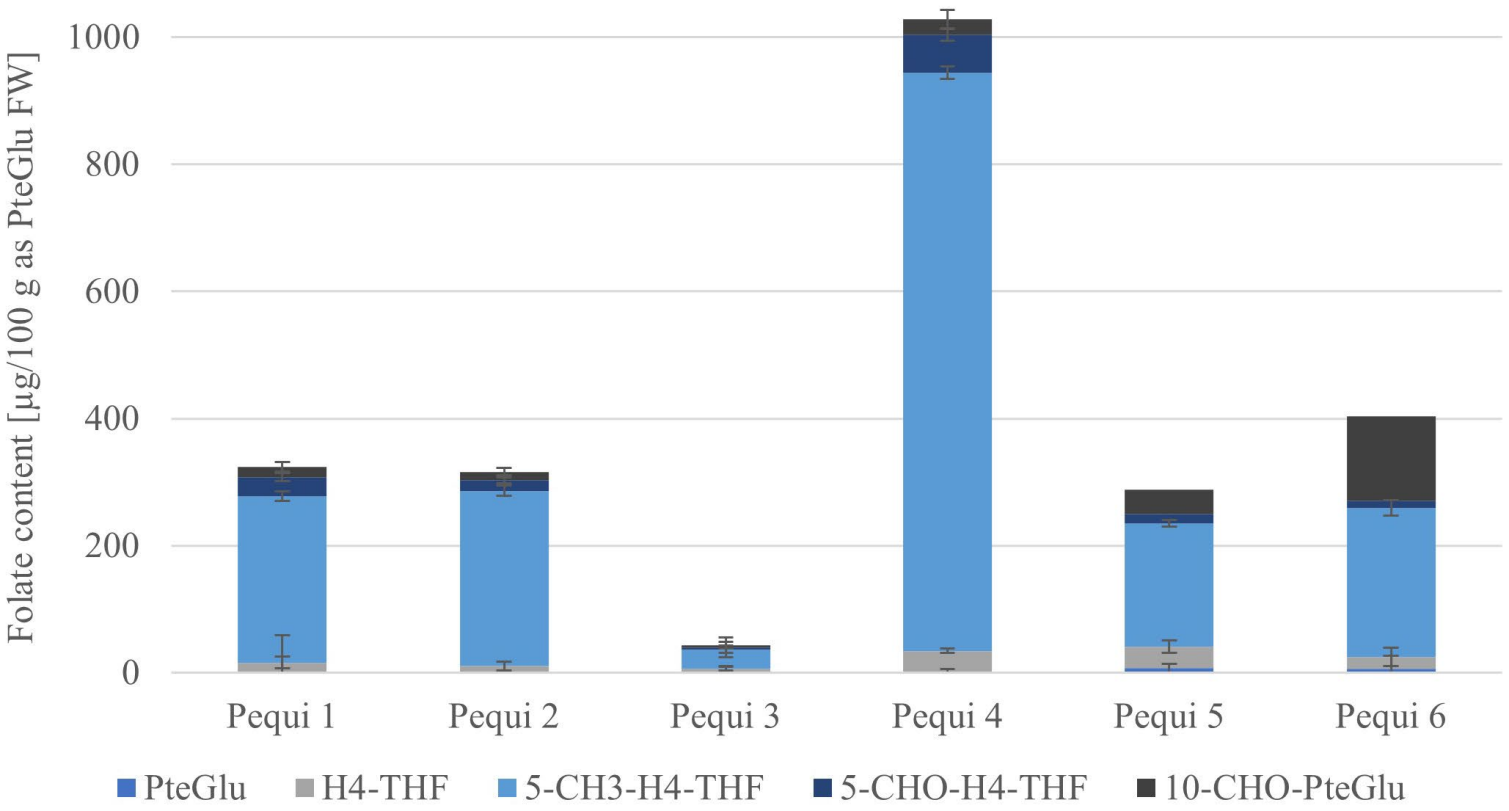
Vitamers distribution of the different pequi samples, calculated as PteGlu in [μg/100 g], based on fresh weight (FW), error bars show relative standard deviation.

The vitamer distribution of each sample is also shown in Figure 2. The folate vitamer 5-CH_3_-H_4_folate was the main folate form in most of the samples. This was expected as it is known to be the main vitamer in most fruits (22). However, in Jenipapo (peel and pulp) and Taioba leaves, the main folate vitamer was 10-CHO-PteGlu. Unexpectedly, the main vitamer of Jatoba (peel) was PteGlu. This finding may be attributed to potential artifacts arising from the sampling or extraction procedures, as this compound lacks bioactivity and does not appear to serve any purpose within the plant. Despite the same sample collection and extraction were applied to each plant, the elevated levels of PteGlu observed in the Jatoba still need to be explored. In contrast, the prevalent vitamer observed in Ora-pro-nóbis was identified as 5-CHO-H_4_folate. This particular vitamer has been documented in other fruits and fermentations, although its exact role in this context has yet to be elucidated. Further research is warranted to comprehend the significance of its abundance in fruits in general (22–24).

### 3.3. Pequi

The outstanding fruit (see Figure 3) in this study was the pequi with folate values of 323 ± 7.02 (pequi 1) and 316 ± 7.09 μg/100 g (pequi 2) fresh weight basis for the external and internal pulp, respectively. However, only the inner pulp is consumed as food (3). This species contains more than 30% of the nutrient reference value of 200 μg/100 g, so it may be labeled “rich in folates” (25). Folate bioavailability depends on several other factors, such as vitamer distribution and cellular structures of the plants (26). Still, nevertheless, the pequi is a promising candidate to meet the daily requirements with the consumption of about 65 g pulp per day.

To verify these results (see Supplementary material Table 3), we were able to analyze four other pequi internal pulp samples. The four additional samples measured showed a wide range of folate content varying from 42.13 ± 5.01 (sample pequi 3) to 986.82 ± 7.95 μg/100 g FW (sample pequi 4). So, one pequi had a 23 times higher folate content than the other, meaning that one would have to eat 200 g of pequi 3 to cover your daily folate reference dose but only 10 g of pequi 4. The samples from the same specimen as pequi sample 4 were more or less in line with the first two analyzed pequis 287.55 ± 4.73 μg/100 g and 403.16 ± 3.62 μg/100 g FW. Therefore, the high value of the pequi 4 with almost 1,000 μg/100 g could not be confirmed with the other new harvest of the same specimen.

It is often the case that different varieties of fruits from various cultivation sites show a great variability in folate content or other nutrient factors. For example, different durian, mango, and guava varieties have been studied. These showed folate values ranging from 307 and 439 μg/100 g FW for durian (27) and folate contents in mangos from 53.7 to 67.7 μg/100 g FW (22), respectively 60.0 and 138 μg/100 g FW (28). The guavas revealed total folate contents from 28.9 to 41.5 μg/100 g FW (22), respectively 49.0 and 211 μg/100 g FW (28). As can be seen from these values, the folate content has been reported to vary by a factor of five. Therefore, some differences between fruit varieties can be expected, but no fruit showed such high differences as the pequi in our study. Pequi 3 is a sample of commercial fruits from Goiás state which were peeled and stored under refrigeration, and pequi 4 fruits were collected in the area of Araraquara city (São Paulo state), processed (separation of peel and pulp) just a few hours after the collection and stored at - 80°C. Thus, geographical location, genetics, maturation stage of the fruits, and storage may cause these differences.

To confirm the high folate content in the freeze-dried samples, we analyzed commercially available pequi samples: a crème (Creme de Pequi), one sauce (Molho de Pequi), and canned pulp fruit pieces. The latter was preserved with vinegar, benzoate, and citric acid. The processed pequis showed highly different results than the fresh and freeze-dried samples, with a maximum of 4.13 μg/100 g FW for the canned fruit and 0.11 and 0.24 μg/100 g FW for the sauce and crème, respectively. Consequently, we investigated the production process and concluded that folate degradation arises due to the harsh processing conditions. For the bottled pulp fruit pieces, the standard production process includes the removal of the peels and storage in a 12% sodium chloride aqueous solution for an undetermined period. Before filling into the final packaging, the fruits are washed with water, which can also lead to losses due to the water solubility of folates, and the bottles are filled with vinegar, benzoate, and citric acid for their preservation. For the crème, the peels are removed from the fruits, sterilized, in which folates can be reduced through heat treatment and released into the cooking water and ground with vinegar to form a cream. Since it is generally known that folates are unstable to very basic and acidic conditions and sensitive to temperatures, the low folate content of these samples is not surprising when considering the processing conditions (8).

### 3.4. Folate content of other (subtropical) fruits

In 2019 Striegel et al. investigated indigenous fruits and vegetables from tropical or subtropical areas, like jackfruit (Moraceae, *Artocarpus heterophyllus* Lam.) with an Indian origin or the Chinese jujube (Rhamnaceae, *Ziziphus jujuba* Mill.), mainly coming from China. The main vitamer in this sample set was 5-CH_3_-H_4_folate, except from salak (Arecaceae, *Salacca zalacca* (Gaertn.) Voss) and one of the horned melon samples (Cucurbitaceae, *Cucumis metuliferus* E.Mey. ex Schrad.), which showed high values for 5-CHO-H_4_folate and tamarind (Fabaceae, *Tamarindus indica* L.) with 10-CHO-PteGlu as the main vitamer. The overall folate content varied from 7.82 ± 0.17 μg/100 g FW (horned melons) to 271 ± 3.64 μg/100 g FW (yellow passionfruit), which covers nearly the same range as our PANC samples from Brazil (22).

The analyzed fruit pequi is typically grown in the Cerrado region of Curvelo, Minas Gerais in Brazil, a region distinguished for its diverse plant life. Another fruit from the same area with a relatively high folate content of around 100 μg/100 g, FW primarily in the form of 5-CHO-H_4_folate, is the mangaba (*Hancornia speciosa* Gomes) (23).

Also, the date palm fruits, an important commercial crop cultivated in Arab countries, have been found to contain a substantial folate content, ranging from 191 to 301 μg/100 g FW with 5-CHO-H_4_folate as the main vitamer, constituting over 90% of the total folate content (24).

A comprehensive study conducted by Akilanathan et al. (28) on the total folate content of subtropical fruits revealed a wide range of folate content on a fresh weight matter, spanning from 9 to 237 μg/100 g. The palmyra fruit exhibited a folate content of 103 μg/100 g, while jamun displayed 167 μg/100 g, figs contained 149 μg/100 g, and custard apple showed 237 μg/100 g. Similarly, temperate fruits were also analyzed by Akilanathan et al. (28). Plum displayed a folate content of 328 μg/100 g, cherry contained 73 μg/100 g, and pear exhibited folate contents from 84 to 147 μg/100 g (28).

These findings demonstrate that (subtropical) fruits can be considered relatively good sources of folate, with variations depending on the specific variety and type. To get more insights into the macronutrients of some of the analyzed PANCs, we determined the centesimal composition. The results can be found in the Supplementary Table 4.

## 4. Conclusion

The study conducted on PANCs revealed valuable insights into the folate content and vitamer distribution of various fruits and leaves. The results showed that half of the analyzed PANCs had relatively low folate content, less than 33 μg/100 g based on fresh weight (FW). However, the standout fruit regarding folate content was pequi, with its internal and external pulp containing more than 300 μg/100 g FW. Similarly, Taioba leaves showed a notable folate content of 185 μg/100 g FW.

Apart from them, the fruit Oiti can be labeled as “rich in folates” with a total folate content of 79 μg/100 g (FW), which proves them to be a promising candidate to address the problem of vitamin deficiency among people living in these areas. The main folate vitamer in most samples was 5-CH_3_-H_4_folate, as expected, considering its prevalence in most fruits. However, Jenipapo (peel and pulp) and Taioba leaves exhibited 10-CHO-PteGlu as the main vitamer.

The study also highlighted that folate content can significantly vary among different fruit varieties, with variations of up to fivefold reported for fruits like durian, mangoes, and guavas. However, none displayed as extreme differences as pequi did in this study.

The validation of the 10-CHO-PteGlu vitamer with reliable and sensitive results in line with the other four validated vitamers in the SIDA method was successful. This enables us to evaluate the folate content in food and other crops even more accurately, especially when the 10-CHO-PteGlu is the main vitamer, as shown in the sample set of PANCs with the samples of Jenipapo and Taioba.

## Data availability statement

The original contributions presented in the study are included in the article/Supplementary material, further inquiries can be directed to the corresponding author.

## Author contributions

AS, BM, and CP selected the species and collected the plant material. BM and CP collected and processed the plant material. LO and MR conceived and designed the experiments. LO performed the folate experiments and analyzed the data. KS and MS conceived and designed the centesimal analysis experiments. MR, VG, and J-PK contributed reagents and materials. MR analyzed the tools. MR, LO, BM, and AS wrote the paper. All authors have read, reviewed, and edited the manuscript and agreed to the published version of the manuscript.

## Funding

This study was partly funded in Brazil by Coordenação de Aperfeiçoamento de Pessoal de Nível Superior-Brasil (CAPES, Finance Code 001) and National Institute of Science and Technology on Biodiversity and Natural Products-INCT-BioNat (CNPq and FAPESP, Grant #465637/2014-0).

## Conflict of interest

RB, VG, and J-PK were employed by Merck & Cie KmG.

The remaining authors declare that the research was conducted in the absence of any commercial or financial relationships that could be construed as a potential conflict of interest.

## Publisher’s note

All claims expressed in this article are solely those of the authors and do not necessarily represent those of their affiliated organizations, or those of the publisher, the editors and the reviewers. Any product that may be evaluated in this article, or claim that may be made by its manufacturer, is not guaranteed or endorsed by the publisher.
