# Supplementary material for: An improved folate stable isotope dilution assay of unexploited food sources from Brazil
Source: Front Nutr. 2023 Sep 5;10:1252497. doi: 10.3389/fnut.2023.1252497 (PMC10508286; doi:10.3389/fnut.2023.1252497)
Supplement: Supplementary file 1 [file Data_Sheet_1.docx]

***Supplementary Material***

# Supplementary Data

**1.1 Centesimal composition analysis of PANCs**

1.1.1 Methodology

The analyzes were conducted according to standardized tests of the Association Official Analytical Chemistry (AOAC), as follows:

- crude proteins were analyzed and quantified by the Kjeldahl method (AOAC, 2006);
- for the determination of total lipids, the Soxhlet method was used (AOAC 2012);
- for the determination of total ash, the muffle method was used (AOAC, 2005);
- total sugar content of samples was determined with 3,5-dinitrosalicylic acid (DNS) (Mañas, Bravo and Saura-Calixto, 1995);
- an enzymatic assay by dialysis was carried out to analyze dietary fibers (Mañas, Bravo and Saura-Calixto, 1995)

1.1.2 Results & Discussion

The centesimal composition of three pequi samples (2, 3 and 4), along with bacupari, canistel, and oiti, was analyzed. The analysis included determining the moisture, total ash, crude proteins, total lipids, dietary fibers (insoluble, soluble, and lignin), carbohydrates, and phenolic compounds. The results are presented in Supplementary Table 4.

The residual moisture in these samples ranged from 3.2% to 7.4%, and the ash content was relatively high, ranging from 1.52% to 3.32% (w/w). This ash content is higher compared to other fruits and vegetables, which typically have ash contents of 0.2% to 0.8% (Pomeranz and Meloan, 1994). Regarding crude protein content, the different pequi varieties exhibited significant differences. Pequi sample 4 had the highest protein content at 25.1%, while sample 3 had the lowest at 7.62%. In terms of total lipids, the pequi samples showed similar levels ranging from 51.4 g/100g DW (pequi 2) to 66.1  /100g DW (pequi 3). On the other hand, canistel and oiti had lower lipid contents of 3.6 g/100g DW and 4.0 g/100g DW, respectively. The analysis of insoluble dietary fibers indicated a balanced distribution among all samples, ranging from 10.4 g/100g DW to 14.5 g/100g DW. However, the pequi samples had notably higher soluble fiber contents, approximately twice as much as the other three samples, ranging from 21.0 g/100g DW to 22.1 g/100g DW. The lignin content was highest in oiti at 25.0 g/100g DW, followed by canistel at 11.2 g/100g DW. The pequi samples had the lowest lignin content in comparison. Regarding carbohydrates, the pequi samples displayed the highest values, averaging at 21.6%. In contrast, bacupari, canistel, and oiti only had an average carbohydrate content of 12.7%. As for phenolic compounds, oiti had the highest content at 13.4%. The pequi samples contained a lower amount of phenolic compounds, ranging from 1.05% to 1.47%.

In summary, the pequi samples exhibited similar contents of lipids, soluble fibers, lignin, carbohydrates, and phenolic compounds. On the other hand, bacupari, canistel, and oiti showed contrasting results for most of these parameters when compared to the pequi samples.

Sources:

AOAC (2005). Official Method 942.05, Determination of Ash in Animal Feed, in Official Methods of Analysis of AOAC International, 18th edition Revision, AOAC International, Gaithersburg, MD, USA.

AOAC (2006). Official Method 990.03, Protein (Crude) in Animal Feed, Combustion Method, in Official Methods of Analysis of AOAC International, 18th edition Revision, AOAC International, Gaithersburg, MD, USA.

AOAC (2012). Official Method 963.15, Fat in cacao products, Soxhlet extraction method, in: Official Methods of Analysis of AOAC International, 19th ed., AOAC International, Gaithersburg, MD, USA.

Mañas, E., & Saura-Calixto, F. (1995). Dietary fibre analysis: methodological error sources. European journal of clinical nutrition, 49 (3), S158–S162.

Pomeranz, Y., Meloan, C.E. (1994). Ash and Minerals. In: Food Analysis. Springer, Boston, MA. https://doi.org/10.1007/978-1-4615-6998-5_35

# Supplementary Tables

**Supplementary Table 1.** Further information on the PANC sample set.

| **Sample** | **Scientific name** | **Part of the fruit** | **Quantities of the same cultivar** | **Origin** |
| --- | --- | --- | --- | --- |
| Bacupari | *Garcinia brasiliensis* Mart. | peel & pulp | 60 units | urban area |
| Canistel | *Pouteria campechiana* (Kunth) Baeni | peel & pulp | 20 units | urban area |
| Jatobá | *Hymenaea courbaril* L. | peel & pulp | 6 units | urban area |
| Jenipapo | *Genipa americana* L. | peel & pulp | 6 units | urban area |
| Oiti | *Licania tomentosa* (Benth) Fritsch | peel & pulp | 6 units | urban area |
| Pequi 1 | *Caryocar brasiliense* Cambess. | external pulp | 6 units | rural area |
| Pequi 2 | *Caryocar brasiliense* Cambess. | inner pulp | 6 units | rural area |
| Ora pro nobis | *Pereskia aculeata* Mill. | leaf | 1,500 kg | urban area |
| Taioba | *Xanthosoma sagittifolium* (L.) Schott | leaf | 1,500 kg | urban area |
| rva-baleeira | *Varronia curassavica* Jacq. | leaf | 10 units | urban area |
| Pequi sample 3 | *Caryocar brasiliense* Cambess. | inner pulp | 6 units | commercial |
| Pequi sample 4 |  | inner pulp | 6 units | urban area |
| Pequi sample 5 |  | inner pulp | 6 units | urban area |
| Pequi sample 6 |  | inner pulp | 6 units | urban area |
| Pequi pieces |  | fruit pieces, bottled | 1 bottle | industrial product |
| Pequi sauce |  | sauce | 1 jar | industrial product |
| Pequi creme |  | creme | 1 jar | industrial product |

**Supplementary Table 2.** MRM scan parameters for the five folate vitamers and the corresponding internal standards.

| **Compound** | **Precursor**  **[m/z]** | **Product**  **[m/z]** | **Dwell time**  **[ms]** | **Q1 Pre**  **bias [V]** | **CE**  **[V]** | **Q3 Pre**  **bias [V]** |
| --- | --- | --- | --- | --- | --- | --- |
| PteGlu | 442.10 | 295.20  176.15  120.15 | 50  50  50 | - 22.0  - 24.0  - 24.0 | - 20.0  - 39.0  - 34.0 | - 22.0  - 18.0  - 28.0 |
| [^13^C_5_]-PteGlu | 447.15 | 295.20  176.25  120.20 | 50  50  50 | - 24.0  - 22.0  - 24.0 | - 18.0  - 38.0  - 45.0 | - 22.0  - 12.0  - 12.0 |
| H_4_Folate | 446.15 | 299.25  166.20  120.20 | 50  50  50 | - 24.0  - 24.0  - 24.0 | - 20.0  - 45.0  - 38.0 | - 22.0  - 18.0  - 24.0 |
| [^13^C_5_]-H_4_Folate | 451.20 | 299.25  166.20  120.20 | 50  50  50 | - 22.0  - 16.0  - 16.0 | - 16.0  - 41.0  - 38.0 | - 22.0  - 18.0  - 14.0 |
| 5-CH_3_-H_4_Folate | 460.10 | 313.30  194.20  180.20 | 50  50  50 | - 26.0  - 14.0  - 24.0 | - 19.0  - 35.0  - 20.0 | - 16.0  - 20.0  - 30.0 |
| [^13^C_5_]-5-CH_3_-  H_4_Folate | 465.20 | 313.25  194.25  180.20 | 50  50  50 | - 24.0  - 24.0  - 24.0 | - 20.0  -30.0  - 35.0 | - 16.0  - 14.0  - 20.0 |
| 5-CHO-H_4_Folate | 474.10 | 327.20  299.20  166.25 | 50  50  50 | - 14.0  - 26.0  - 14.0 | - 20.0  - 32.0  - 44.0 | - 16.0  - 22.0  - 12.0 |
| [^13^C_5_]-5-CHO-  H_4_Folate | 479.15 | 327.20  299.20  166.30 | 50  50  50 | - 14.0  - 24.0  - 14.0 | - 20.0  - 32.0  - 48.0 | - 24.0  - 42.0  - 18.0 |
| 10-CHO-PteGlu | 470.10 | 323.15  295.20  176.20  120.20 | 50  50  50  50 | - 24.0  - 24.0  - 24.0  - 14.0 | - 18.0  - 26.0  - 41.0  - 40.0 | - 10.0  - 20.0  - 20.0  - 14.0 |
| [^13^C_5_]-10-CHO-PteGlu | 475.10 | 323.20  295.15  176.20  120.10 | 50  50  50  50 | - 14.0  - 24.0  - 24.0  - 24.0 | - 18.0  - 24.0  - 40.0  - 41.0 | - 26.0  - 22.0  - 28.0  - 20.0 |

**Supplementary Table 3**. Total folate content and vitamer distribution of various PANCs, calculated as PteGlu in [μg/100 g] on dry and fresh weight basis

| **Sample** | **PteGlu**  **[µg/100 g]** | **H_4_folate**  **[µg/100 g as PteGlu]** | **5-CH_3_-H_4_folate**  **[µg/100 g as PteGlu]** | **5-CHO-H_4_folate**  **[µg/100 g as PteGlu]** | **10-CHO-PteGlu**  **[µg/100 g as PteGlu]** | **Total folate (dry weight basis )**  **[µg/100 g as PteGlu]** | **Total folate (fresh weight basis)**  **[µg/100 g as PteGlu]** |
| --- | --- | --- | --- | --- | --- | --- | --- |
| Bacupari (peel) | 1.18 ± 5.63 | 13.19 ± 4.08 | 68.70 ± 1.56 | 9.78 ± 2.98 | 5.99 ± 10.81 | 98.83 ± 0.91 | **13.74** |
| Bacupari (pulp) | 2.77 ± 5.92 | 2.81 ± 4.93 | 16.99 ± 7.66 | 11.59 ± 9.67 | 17.52 ± 5.25 | 51.68 ± 9.92 | **9.11** |
| Canistel (peel) | < LoQ | 4.72 ± 4.97 | 36.45 ± 4.98 | 4.63 ± 5.18 | 1.36 ± 23.50 | 46.79 ± 3.48 | **18.44** |
| Canistel (pulp) | < LoQ | 8.19 ± 16.29 | 53.11 ± 6.29 | 4.41 ± 4.10 | < LoQ | 66.97 ± 7.27 | **26.38** |
| Jatobá (peel) | 18.27 ± 13.93 | 1.52 ± 8.00 | 7.87 ± 2.02 | 6.68 ± 6.02 | 7.82 ± 11.56 | 42.15 ± 6.38 | **19.18** |
| Jatobá (pulp) | 2.96 ± 3.30 | 2.92 ± 7.88 | 43.73 ± 4.87 | 16.80 ± 6.04 | 4.76 ± 42.02 | 71.17 ± 4.54 | **32.38** |
| Jenipapo (peel) | 15.42 ± 13.12 | < LoQ | 1.30 ± 81.09 | 1.40 ± 2.41 | 216.28 ± 4.72 | 234.61 ± 5.63 | **30.62** |
| Jenipapo (pulp) | 3.63 ± 3.44 | 1.61 ± 5.58 | n.d. | 0.97 ± 3.71 | 39.90 ± 2.28 | 46.10 ± 1.76 | **6.02** |
| Oiti (fruit peel) | < LoQ | 15.16 ± 3.95 | 283.77 ± 5.77 | 8.44 ± 8.14 | 6.42 ± 32.70 | 314.60 ± 5.00 | **78.90** |
| Oiti (pulp) | < LoQ | 13.41 ± 7.87 | 287.70 ± 5.27 | 6.50 ± 9.39 | 6.00 ± 19.97 | 314.19 ± 4.90 | **78.80** |
| Pequi 1  (external pulp - rural area) | 3.39 ± 7.47 | 32.08 ± 9.20 | 588.31 ± 7.21 | 68.23 ± 6.39 | 34.28 ± 7.44 | 726.30 ± 7.02 | **323.35** |
| Pequi 2  (inner pulp - rural area) | n.d. | 23.97 ± 6.96 | 618.89 ± 7.85 | 37.09 ± 4.61 | 29.82 ± 5.76 | 709.78 ± 7.09 | **315.99** |
| Ora pro nobis (leaf) | 3.21 ± 4.37 | 3.50 ± 7.86 | 33.62 ± 7.66 | 96.40 ± 4.92 | 47.28 ± 1.45 | 184.01 ± 1.05 | **21.84** |
| Taioba (leaf) | 15.90 ± 10.94 | 36.27 ± 10.79 | 367.92 ± 9.93 | 232.32 ± 3.96 | 447.99 ± 1.90 | 1100.40 ± 4.56 | **185.80** |
| Erva-baleeira (leaf) | 11.88 ± 7.38 | 30.01 ± 7.09 | 405.81 ± 7.94 | 117.52 ± 11.51 | 156.88 ± 2.50 | 722.09 ± 3.09 | **79.43** |

**Supplementary Table 3.** Extension

| **Sample** | **PteGlu**  **[µg/100 g]** | **H_4_folate**  **[µg/100 g as PteGlu]** | **5-CH_3_-H_4_folate**  **[µg/100 g as PteGlu]** | **5-CHO-H_4_folate**  **[µg/100 g as PteGlu]** | **10-CHO-PteGlu**  **[µg/100 g as PteGlu]** | **Total folate (dry weight basis )**  **[µg/100 g as PteGlu]** | **Total folate (fresh weight basis)**  **[µg/100 g as PteGlu]** |
| --- | --- | --- | --- | --- | --- | --- | --- |
| Pequi sample 3 (commercial inner pulp) | 1.82 ± 9.60 | 11.76 ± 2.58 | 68.31 ± 5.94 | 7.96 ± 15.58 | 6.74 ± 4.85 | 96.59 ± 5.01 | **42.13** |
| Pequi sample 4 (inner pulp - urban area) | 1.86 ± 5.04 | 75.01 ± 3.25 | 2043.24 ± 9.02 | 135.00 ± 10.22 | 53.56 ± 14.88 | 2308.67 ± 7.95 | **986.82** |
| Pequi sample 5 (inner pulp - urban area) | 15.34 ± 6.45 | 76.49 ± 9.28 | 435.83 ± 5.70 | 32.78 ± 7.89 | 85.43 ± 20.35 | 645.88 ± 4.73 | **287.55** |
| Pequi sample 6 (inner pulp - urban area) | 14.03 ± 20.24 | 41.71 ± 14.59 | 526.13 ± 11.91 | 27.22 ± 10.69 | 296.46 ± 17.25 | 905.56 ± 3.62 | **403.16** |
| Pequi – Fruit pieces, bottled | 16.69 ± 5.51 | 2.05 ± 19.61 | < LoQ | 1.14 ± 78.15 | 2.88 ± 1.47 | 22.53 ± 9.07 | **9.35** |
| Pequi - Sauce | < LoQ | < LoQ | < LoQ | < LoQ | < LoQ | < LoQ  (0.24 ± 82.55) | - |
| Pequi - Creme | < LoQ | < LoQ | < LoQ | < LoQ | < LoQ | < LoQ  (0.11 ± 17.87) | - |

**Supplementary Table 4.** Centesimal composition of PANCs.

| ***Scientific names of samples***  **(popular name)** | ***Caryocar brasiliensis* Cambess (pequi 2 - rural)** | ***Caryocar brasiliensis* Cambess (pequi 3 - commercial)** | ***Caryocar brasiliensis* Cambess (pequi 4 - urban)** | ***Garcinia brasiliensis* Mart., (bacupari)** | ***Pouteria campechiana* (Kunth) Baehni (canistel)** | ***Licania tomentosa* (Benth.) Fritsch**  **(oiti)** |
| --- | --- | --- | --- | --- | --- | --- |
| residual moisture [%] | 3.50 ± 0.30 | 3.20 ± 0.60 | 4.30 ± 0.30 | 6.60 ± 1.50 | 7.40 ± 0.10 | 4.80 ± 0.10 |
| ash [%, w/w] | 1.90 ± 0.11 | 2.08 ± 0.06 | 2.32 ± 0.06 | 1.52 ± 0.03 | 1.81 ± 0.02 | 3.32 ± 0.09 |
| protein [%] | 15.29 ± 1.24 | 7.62 ± 1.48 | 25.10 ± 0.92 | 8.59 ± 0.49 | 13.19 ± 1.30 | 15.46 ± 0.45 |
| lipids [%] | 56.7 ± 0.20 | 66.1 ± 0.60 | 51.4 ± 0.60 | 11.5 ± 1.80 | 3.6 ± 1.50 | 4.0 ± 1.60 |
| soluble fiber (SF) [g/100 g DW] | 22.1 ± 3.00 | 21.0 ± 0.90 | 21.7 ± 1.40 | 12.8 ± 0.90 | 13.3 ± 0.20 | 12.1 ± 0.30 |
| insoluble fiber (IF) [g/100 g DW] | 11.0 ± 1.10 | 14.1 ± 1.00 | 14.5 ± 0.20 | 10.5 ± 1.30 | 12.3 ± 0.40 | 10.4 ± 0.80 |
| lignin (L) [g/100 g DW] | 4.5 ± 4.40 | 1.6 ± 0.30 | 2.4 ± 0.30 | 5.7 ± 1.10 | 11.2 ± 0.50 | 25.0 ± 2.70 |
| total fiber (SF+IF+L) [g/100 g DW] | 37.3 ± 8.30 | 36.6 ± 1.90 | 38.5 ± 1.30 | 28.9 ± 1.90 | 36.8 ± 0.70 | 47.5 ± 3.10 |
| carbohydrate [%] | 22.1 ± 3.00 | 21.0 ± 0.90 | 21.7 ± 1.40 | 12.8 ± 0.90 | 13.3 ± 0.20 | 12.1 ± 0.30 |
| phenolic compounds GAE [%] | 1.14 ± 0.10 | 1.47 ± 0.20 | 1.05 ± 0.30 | enp | 4.86 ± 0.70 | 13.4 ± 2.40 |

GAE = gallic acid equivalent DW = Dry weight enp = experiment not performed
